# Comparative proteome analysis identified CD44 as a possible serum marker for docetaxel resistance in castration‐resistant prostate cancer

**DOI:** 10.1111/jcmm.17141

**Published:** 2021-12-30

**Authors:** Dávid Keresztes, Anita Csizmarik, Nikolett Nagy, Orsolya Módos, Tamás Fazekas, Thilo Bracht, Barbara Sitek, Kathrin Witzke, Martin Puhr, Sabina Sevcenco, Gero Kramer, Shahrokh Shariat, Zsófia Küronya, László Takács, Ilona Tornyi, József Lázár, Boris Hadaschik, András Lászik, Miklós Szűcs, Péter Nyirády, Tibor Szarvas

**Affiliations:** ^1^ Department of Urology Semmelweis University Budapest Hungary; ^2^ Medical Faculty Medizinisches Proteom‐Center Ruhr‐University Bochum Bochum Germany; ^3^ Department of Anesthesia Intensive Care Medicine and Pain Therapy University Hospital Knappschaftskrankenhaus Bochum Bochum Germany; ^4^ Center for Protein Diagnostics Medical Proteome Analysis Ruhr‐University Bochum Bochum Germany; ^5^ Department of Urology Medical University of Innsbruck Innsbruck Austria; ^6^ Department of Urology Donauspital Vienna Austria; ^7^ Department of Urology Medical University of Vienna Vienna Austria; ^8^ Department of Genitourinary Medical Oncology and Clinical Pharmacology National Institute of Oncology Budapest Hungary; ^9^ Department of Human Genetics Faculty of Medicine University of Debrecen Debrecen Hungary; ^10^ Biosystems International Kft. Debrecen Hungary; ^11^ Department of Urology University of Duisburg‐Essen Essen Germany; ^12^ Department of Forensic and Insurance Medicine Semmelweis University Budapest Hungary

**Keywords:** biomarker research, castration‐resistant prostate cancer, comparative proteome analysis, docetaxel resistance, LC‐MS/MS

## Abstract

Baseline or acquired resistance to docetaxel (DOC) represents a significant risk for patients with metastatic prostate cancer (PC). In the last years, novel therapy regimens have been approved providing reasonable alternatives for DOC‐resistant patients making prediction of DOC resistance of great clinical importance. We aimed to identify serum biomarkers, which are able to select patients who will not benefit from DOC treatment. DOC‐resistant PC3‐DR and DU145‐DR sublines and their sensitive parental cell lines (DU145, PC3) were comparatively analyzed using liquid chromatography‐coupled tandem mass spectrometry (LC‐MS/MS). Results were filtered using bioinformatics approaches to identify promising serum biomarkers. Serum levels of five proteins were determined in serum samples of 66 DOC‐treated metastatic castration‐resistant PC patients (mCRPC) using ELISA. Results were correlated with clinicopathological and survival data. CD44 was subjected to further functional cell culture analyses. We found at least 177 two‐fold significantly overexpressed proteins in DOC‐resistant cell lines. Our bioinformatics method suggested 11/177 proteins to be secreted into the serum. We determined serum levels of five (CD44, MET, GSN, IL13RA2 and LNPEP) proteins in serum samples of DOC‐treated patients and found high CD44 serum levels to be independently associated with poor overall survival (*p *= 0.001). In accordance, silencing of CD44 in DU145‐DR cells resulted in re‐sensitization to DOC. In conclusion, high serum CD44 levels may help identify DOC‐resistant patients and may thereby help optimize clinical decision‐making regarding type and timing of therapy for mCRPC patients. In addition, our in vitro results imply the possible functional involvement of CD44 in DOC resistance.

## 1 INTRODUCTION

Prostate cancer (PC) is the second most common solid cancer and the fifth leading cause of cancer mortality among men worldwide.^1^ Docetaxel (DOC) chemotherapy is one of the standard first‐line therapy options for metastatic castration‐resistant PC (mCRPC), but about half of the patients show initial resistance, and most of the patients will develop acquired resistance to DOC.^2^, ^3^, ^4^

Despite significant progress in the understanding of the molecular background of DOC resistance, prediction of DOC treatment remains an unmet clinical need. Thus, our purpose was to gain insight into the molecular mechanisms of DOC resistance in PC and to identify novel therapy‐predictive biomarkers and to find potential therapy targets. In order to identify differently expressed proteins between DOC‐resistant *vs*. DOC‐sensitive PC cell lines, we performed a comparative proteome analysis. Potentially secreted proteins were filtered by using bioinformatics approaches. Selected proteins were quantitatively analyzed in serum samples of mCRPC patients before DOC chemotherapy. Serum concentrations were correlated with clinical and follow‐up data in order to determine their value for the prediction of response and survival of DOC‐treated patients. Finally, we performed functional *in vitro* analyses by knocking down target proteins in PC cell lines and assessed its effect on DOC‐sensitivity.

## 2 MATERIALS AND METHODS

### 2.1 Culturing and LC‐MS/MS analysis of DOC‐sensitive vs. ‐resistant PC cell lines

DU145 and PC3 human PC cell lines were purchased from ATCC (Rockville, MD), and their DOC‐resistant sublines (DU145‐DR, PC3‐DR) were developed by adding increasing concentrations of DOC (Sigma‐Aldrich, St. Louis, MO, USA) up to the maintenance concentration of 12.5 nmol/L as described earlier.^5^

Proteome analyses were performed using the LC‐MS/MS technique in order to identify differently abundant proteins between the DOC‐sensitive (PC3, DU145) versus DOC‐resistant (PC3‐DR, DU145‐DR) cell lines (see Supplementary Methods).

### 2.2 Biomarker selection

Proteins quantified by LC‐MS/MS were tested for differential abundance between parental and DOC‐resistant PC cell lines. Proteins quantify with minimum two unique peptides, and those passing the applied significance thresholds (FDR‐corrected *p*‐value ≤0.05, fold change ≥2) were considered for further analysis. Bioinformatics methods were applied to identify potentially secreted proteins (see Supplementary Methods and Figure S1).

### 2.3 Patient cohort and samples

Serum samples were collected between 01/2014 and 03/2018 from 66 consenting mCRPC patients directly before treatment with first‐line DOC.

PSA response was defined as at least 50% PSA decline from baseline during the first chemotherapy series. The Institutional Ethics Committee approved the study protocol (TUKEB: 55/2014). The main endpoint of the analysis was overall survival (OS).

### 2.4 Serum ELISA analyses

Serum concentrations of CD44, HGFR and IL13RA2 proteins were quantified using DuoSet ELISA kits (R&D Systems, MN, USA), while LNPEP and GSN levels were determined by using ELISA kits by Nordic and LSBio (Nordic BioSite, Täby, Sweden; LSBio, WA, USA, respectively).

### 2.5 Functional experiments

Based on the results of the ELISA analysis, we selected CD44 for further functional experiments. CD44 was knocked down in PC3‐DR and DU145‐DR cell lines by using the siRNA technique. Knock down was confirmed by Western blot and ELISA analyses, while cell cycle analysis was performed by flow cytometry. For RT‐qPCR and Western blot analysis methods as well as for statistical methods please see Supplementary Methods.

## 3 RESULTS

### 3.1 Differentially expressed proteins between DOC sensitive vs. ‐resistant PC cells

Proteome analyses revealed 685 (DU145 vs. DU145‐DR) and 248 (PC3 vs. PC3‐DR) significantly differentially abundant (either increased or decreased) proteins of which 146 and 31 were at least 2‐fold upregulated in DU145‐DR and PC3‐DR cell lines, respectively (see Figure S2).

We found ABCB1, SYPL1 and HSPB1 to be consequently highly abundant in both DOC‐resistant sublines. The 10 most upregulated proteins in resistant cells are listed in Table S1.

Out of the 146 (DU145‐DR) and 31 (PC3‐DR) upregulated proteins, our prediction algorithm for secreted proteins identified five (CD44, GSN, CALU, COASY and HBS1L) and six (IL13RA2, COL6A1, MET, AUP1, ERAP1 and LNPEP) potentially secreted proteins, respectively. Five of these proteins (CD44, LNPEP, GSN, IL13RA2 and MET) were selected for ELISA analysis in clinical serum samples.

### 3.2 Correlation of serum marker levels with clinicopathological parameters and survival

**FIGURE 1:**
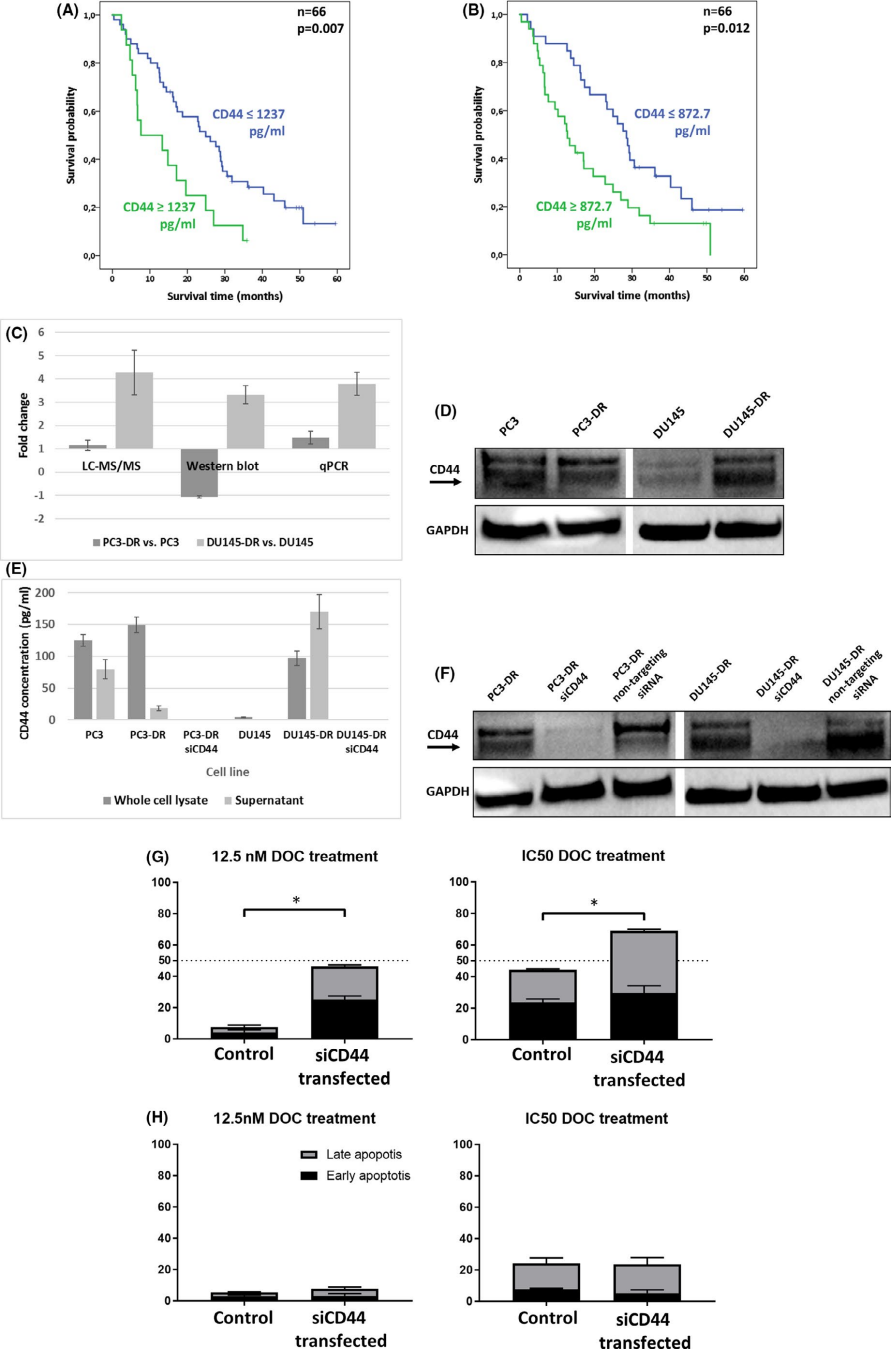
Experimental Results. (A–B) Kaplan–Meier Plots. Survival analysis showed that elevated pre‐treatment serum CD44 levels (A: 1237 pg/ml ‐ upper 25%; B: 872.7 pg/ml ‐ median) are associated with poor survival in mCRPC patients who underwent DOC treatment. (C–D) Gene and protein expression levels of CD44 in DOC‐sensitive and ‐resistant PC cell lines. The expression values determined by the three different methods are considerably consistent (C). Similar to the findings by the LC‐MS/MS analysis, which found 4.28‐fold and 1.15‐fold overexpression of CD44 in DU145‐DR and PC3‐DR cells, our real‐time qPCR‐based method found 3.79‐ and 1.48‐fold overexpression at the mRNA level, and our Western blot analyses assessed 3.32‐fold upregulation and −1.07‐fold downregulation at the protein level, respectively (C–D). Fold changes in Western blot experiments were assessed by densitometry (D). CD44 fold change value of DU145‐DR cells measured by Western blot was divided by 3 on the bar chart (C) for better visualization. (E–F) Monitoring of siRNA‐mediated gene silencing in cell lines by ELISA (E) and Western blot (F). Presence of CD44 was detectable by ELISA in cell lysates, and in their supernatant as well. After gene silencing, the level of CD44 was undetectable by ELISA in whole cell lysates and in their supernatant as well. In CD44‐silenced cells, CD44 was undetectable by ELISA neither in the cell lysate nor in the supernatant (E). Transfection with non‐targeting siRNA had no effect on the protein expression of CD44 (F). Whole cell lysate and supernatant values are divided by 250 and 2, respectively, on the bar charts (E) for better visualization. CD44 and GAPDH bands of PC3‐DR and DU145‐DR cells from the very same Western blot were repeatedly used in plots D and F in order to provide a direct comparison between sensitive vs. resistant (D) and CD44 knockout vs. control cell lines (F). (G–H). Apoptosis analysis by flow cytometry was performed on both DOC‐resistant cell lines. Regarding the experiments conducted on DU145‐DR cells, (G) we could detect significant changes (*p < 0.01). The mean rate of the living DU145‐DR cells due to siCD44 transfection changed from 88.59% to 47.57% (under 12.5 nM DOC treatment), and from 50.07% to 27.22% (IC50 = 100 nM DOC). Meanwhile, the mean rate of the apoptotic cells in the population increased from 7.50% to 46.30% (12.5 nM DOC), and from 44.11% to 65.92% (IC50 = 100 nM DOC). The same experiments with PC3‐DR cells did not show similar changes (H; IC50 DOC concentration for PC3‐DR cells: 200 nM)

The main patients’ and follow‐up characteristics are given in Table 1. Two proteins, GSN and IL13RA2, were undetectable in serum samples, while LNPEP showed detectable signals only in 15 of 53 samples; therefore, for LNPEP, results were dichotomized as positive vs. negative. We found no significant correlations between the assessed marker levels (MET, CD44, LNPEP and PSA) and clinicopathological parameters (see Table S2). Univariate Cox analysis showed ECOG performance status (>0) and PSA response to be significantly associated with shorter OS (*p *= 0.039, *p *= 0.001, respectively). In addition, CD44 levels greater than the median and upper 25% were correlated with poor OS (*p *= 0.014, *p *= 0.009, respectively) (see Table S3). Multivariate analysis revealed high CD44 serum level (upper 25%) to be independently associated with poor OS (*p *= 0.016) (see Figure 1A,B and Table S4).

**TABLE 1:** Clinical characteristics of patients who underwent serum ELISA analyses for CD44, MET, IL13RA2, GSN and LNPEP

	CD44, MET, IL13RA2	LNPEP, GSN^a^
Total number of patients	66	53
Age at baseline median (range)	71 (44–86)	71 (44–86)
ECOG PS at enrollment		
0	38	30
1	22	19
2	5	3
unknown	1	1
Bone metastasis	64	51
Lymph node metastasis. (>2 cm)	29	21
Soft tissue lesions (lung/liver)	14	10
Previous prostatectomy	12	10
Previous radiation	7	5
PSA at baseline (ng/ml)	87.8 (2.6–7312.0)	88.9 (7.9–7312.0)
any PSA decline (yes / no / N.A.)	46 / 11 / 9	36 / 10 / 7
PSA decline 30%	36	27
PSA decline 50%	31	23
PSA decline 90%	16	10
PSA progression on DOC (yes/no)	15 / 31	14 / 22
Rad. progression on DOC (yes / no / N.A.)	24 / 18 / 24	22 / 15 / 16
Number of patients died /alive	42 / 24	38 / 15
Follow‐up time in months median	19	19

Abbreviation: ECOG PS, Eastern Cooperative Oncology Group Performance Status.

^a^ For the LNPEP and GSN analysis, because of limited sample volumes in some cases, only 53 samples were available.

### 3.3 **CD44** **knockdown by siRNA and its effect on cell viability and cell cycle**

CD44 mRNA and protein levels as determined by RT‐qPCR and Western blot analysis correlated well with the protein levels determined by LC‐MS/MS analyses (see Figure 1C,D). The siRNA transfection successfully silenced CD44 expression in DU145‐DR cells and completely in PC3‐DR cells according to Western blot and ELISA measurements (see Figure 1E,F).

Apoptosis analysis performed by flow cytometry using double staining (propidium iodide and Annexin V) revealed enhanced DOC‐sensitivity in CD44 silenced DU145‐DR cells compared to the controls under maintenance (12,5 nM) and also IC50 dose (100 nM) of DOC treatment as the rate of the living cells of the population decreased while the ratio of the apoptotic cells increased (see Figure 1G and Figure S3A,C). Similar experiments on PC3‐DR cells did not reveal significant changes in their DOC resistance (see Figure 1H and Figure S3B,D).

## 4 DISCUSSION

Resistance of mCRPC to DOC remains incompletely understood. Currently known DOC resistance mechanisms include the overexpression of the drug transporter protein ABCB1 (also known as MDR1),^6^, ^7^ inflammatory proteins (IL‐6, YKL‐40 and CCL2)^8^, ^9^, ^10^, ^11^ and the transcription factor ERG, which is frequently (>50%) upregulated due to a chromosomal translocation resulting in TMPRSS‐ERG gene fusion.^12^, ^13^ Further studies pointed at the correlation between the presence of an androgen receptor splice variant (AR‐V7) and taxane resistance.^14^, ^15^ These results suggest that DOC resistance is molecularly divergent, and presumably more than one mechanism may contribute to therapy insensitivity.

The rapidly improving treatment landscape of mCRPC provides reasonable alternatives (cabazitaxel, abiraterone, enzalutamide, alpharadin, olaparib, rucaparib, pembrolizumab and ^177^lutetium‐PSMA) for DOC‐resistant patients. This development makes therapeutic decision‐making increasingly complex. Lacking predictive and prognostic biomarkers, the optimal treatment sequence today does not account for molecular features of the tumour.

In the present study, using a hypothesis‐free comparative proteomic analysis in DOC‐resistant vs. sensitive parental PC cell lines, we identified a large number of proteins potentially involved in DOC resistance. These data provide a solid base for further research towards the elucidation of DOC resistance (see Table S5,S6). Five proteins (MET, CD44, LNPEP, GSN and IL13RA2) were selected for quantitative analysis in serum samples of DOC‐treated mCRPC patients. This analysis revealed high baseline serum CD44 levels as an independent predictor of shorter survival in DOC‐treated patients. In accordance, our functional in vitro analysis showed that knockdown of CD44 re‐sensitized resistant DU145‐DR cells to DOC. These promising results warrant for further validation in an independent patient cohort. In this study, we focused on secreted proteins, but in the Supplementary Material, we provide a more detailed discussion on further promising marker candidates (see Supplementary Discussion).

## CONFLICT OF INTEREST

The authors declare no conflict of interest.

## AUTHOR CONTRIBUTIONS

**Dávid Keresztes:** Conceptualization (supporting); Data curation (lead); Formal analysis (lead); Funding acquisition (supporting); Investigation (lead); Methodology (lead); Project administration (lead); Software (supporting); Visualization (lead); Writing – original draft (lead). **Anita Csizmarik:** Data curation (equal); Formal analysis (supporting); Project administration (lead); Writing – review & editing (supporting). **Nikolett Nagy:** Data curation (equal); Formal analysis (supporting); Visualization (supporting); Writing – review & editing (supporting). **Orsolya Módos:** Data curation (equal); Formal analysis (equal); Writing – review & editing (supporting). **Tamás Fazekas:** Data curation (lead); Writing – review & editing (supporting). **Thilo Bracht:** Conceptualization (supporting); Data curation (supporting); Formal analysis (equal); Investigation (equal); Methodology (supporting); Resources (supporting); Software (supporting); Validation (supporting); Writing – review & editing (supporting). **Barbara Sitek:** Conceptualization (supporting); Formal analysis (equal); Funding acquisition (supporting); Investigation (equal); Methodology (supporting); Resources (supporting); Software (supporting); Validation (supporting); Visualization (supporting); Writing – review & editing (supporting). **Kathrin Witzke:** Conceptualization (supporting); Formal analysis (lead); Funding acquisition (supporting); Investigation (equal); Methodology (equal); Resources (supporting); Software (supporting); Validation (supporting); Visualization (equal); Writing – review & editing (supporting). **Martin Puhr:** Funding acquisition (supporting); Resources (lead); Visualization (supporting); Writing – review & editing (supporting). **Sabina Sevcenco:** Data curation (lead); Resources (lead); Writing – review & editing (supporting). **Gero Kramer:** Data curation (lead); Resources (lead); Writing – review & editing (supporting). **Shahrokh Shariat:** Data curation (supporting); Resources (equal); Writing – review & editing (supporting). **Zsófia Küronya:** Data curation (equal); Visualization (supporting); Writing – review & editing (supporting). **László Takács:** Data curation (equal); Software (equal); Writing – review & editing (supporting). **Ilona Tornyi:** Data curation (equal); Software (equal); Writing – review & editing (supporting). **József Lázár:** Data curation (equal); Software (equal); Writing – review & editing (supporting). **Boris Hadaschik:** Conceptualization (equal); Visualization (supporting); Writing – review & editing (equal). **András Lászik:** Resources (equal); Writing – review & editing (supporting). **Miklós Szűcs:** Data curation (equal); Resources (equal); Writing – review & editing (supporting). **Péter Nyirády:** Conceptualization (equal); Supervision (lead); Visualization (supporting); Writing – review & editing (equal). **Tibor Szarvas:** Conceptualization (lead); Data curation (equal); Formal analysis (supporting); Funding acquisition (lead); Investigation (supporting); Methodology (supporting); Project administration (supporting); Supervision (lead); Visualization (supporting); Writing – original draft (lead); Writing – review & editing (lead).

## Supporting information

Fig S1

Fig S2

Fig S3

Table S1

Table S2

Table S3

Table S4

Supplementary Material

Supplementary Material

## Data Availability

The data that supports the findings of this study are available in the Supplementary Material of this article.
