# Supplementary material for: Comparative proteome analysis identified CD44 as a possible serum marker for docetaxel resistance in castration‐resistant prostate cancer
Source: J Cell Mol Med. 2021 Dec 30;26(4):1332–7. doi: 10.1111/jcmm.17141 (PMC8831956; doi:10.1111/jcmm.17141)
Supplement: Supplementary file 1 — Fig S1 [file JCMM-26-1332-s009.docx]

**
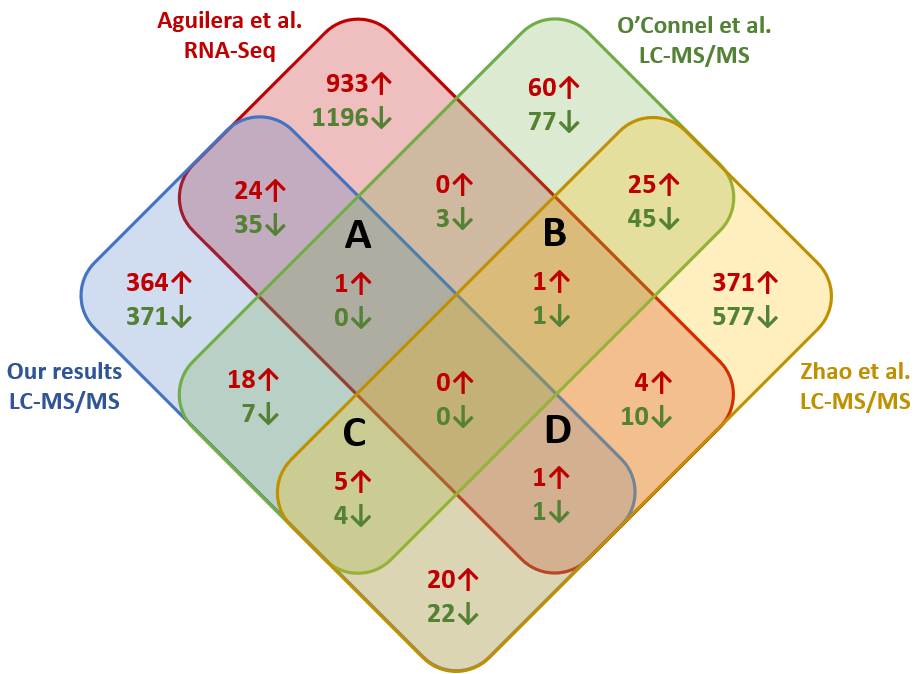
**

**Supplementary figure 1.** **Venn diagram comparison of differential expressed genes/proteins identified in the present work and those in previous studies.** Cross-reference analysis was performed with three published data sets based on comparative analyses of DOC-sensitive and resistant prostate cancer cell lines. Two of the three analyses used LC-MS/MS technique for comparison^1, 2^ while one of them is based on global gene expression array data (mRNA)^3^. Red numbers represent upregulated proteins, while green numbers indicate the downregulated ones. The intersections indicated with letters contain those proteins which were found to be up- or downregulated according to at least three independent studies.

A - Upregulation: ANXA6

B - Upregulation: PYGL; Downregulation: EFHD2

C - Upregulation: ANXA3, SND1, FLNC, NAMPT, ACTN1; Downregulation: PDIA3, OAT, LDHA, FLNB

D - Upregulation: PYGB; Downregulation: CKAP4

References:

1. O'Connell K, Prencipe M, O'Neill A, et al. The use of LC-MS to identify differentially expressed proteins in docetaxel-resistant prostate cancer cell lines. *Proteomics*. 2012;12(13):2115-26.

2. Zhao L, Lee BY, Brown DA, et al. Identification of candidate biomarkers of therapeutic response to docetaxel by proteomic profiling. *Cancer Res*. 2009;69(19):7696-703.

3. Marin-Aguilera M, Codony-Servat J, Kalko SG, et al. Identification of docetaxel resistance genes in castration-resistant prostate cancer. *Mol Cancer Ther*. 2012;11(2):329-39.
