# Supplementary material for: Comparative proteome analysis identified CD44 as a possible serum marker for docetaxel resistance in castration‐resistant prostate cancer
Source: J Cell Mol Med. 2021 Dec 30;26(4):1332–7. doi: 10.1111/jcmm.17141 (PMC8831956; doi:10.1111/jcmm.17141)
Supplement: Supplementary file 2 — Fig S2 [file JCMM-26-1332-s006.docx]

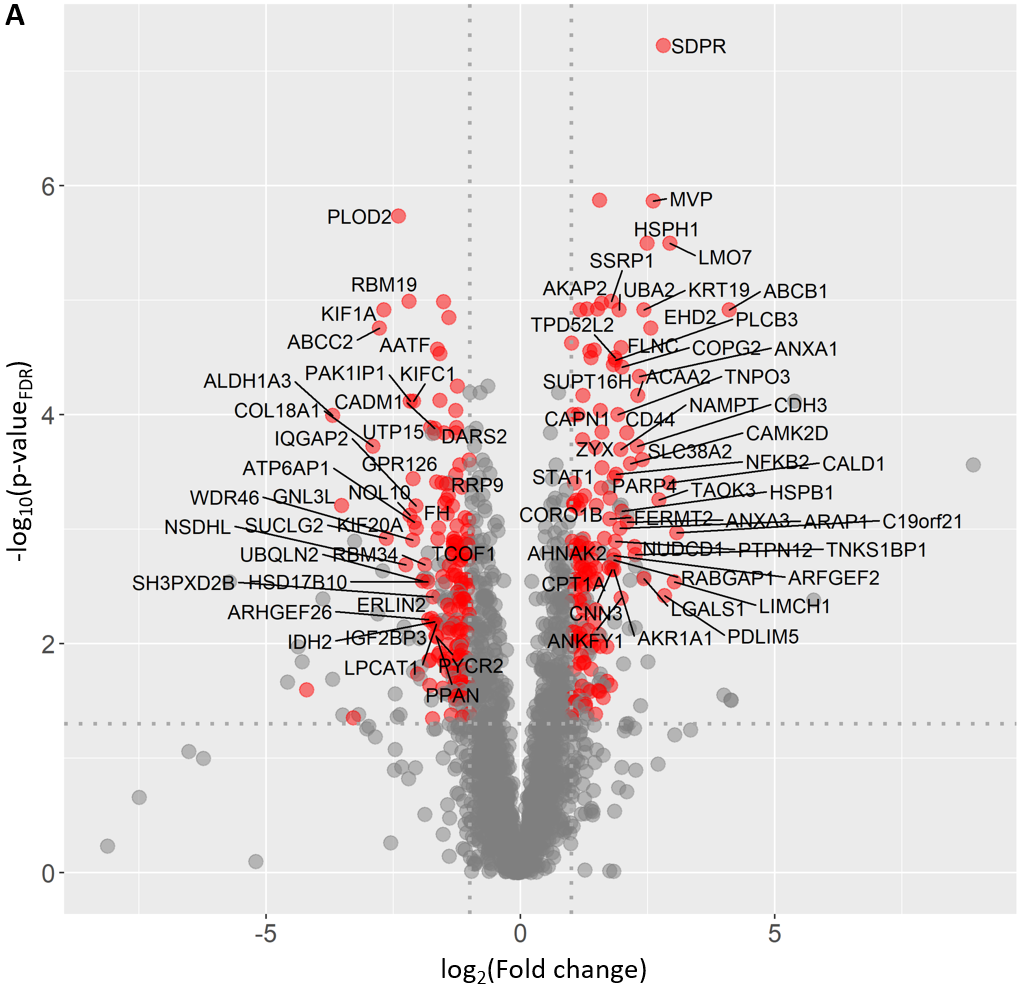


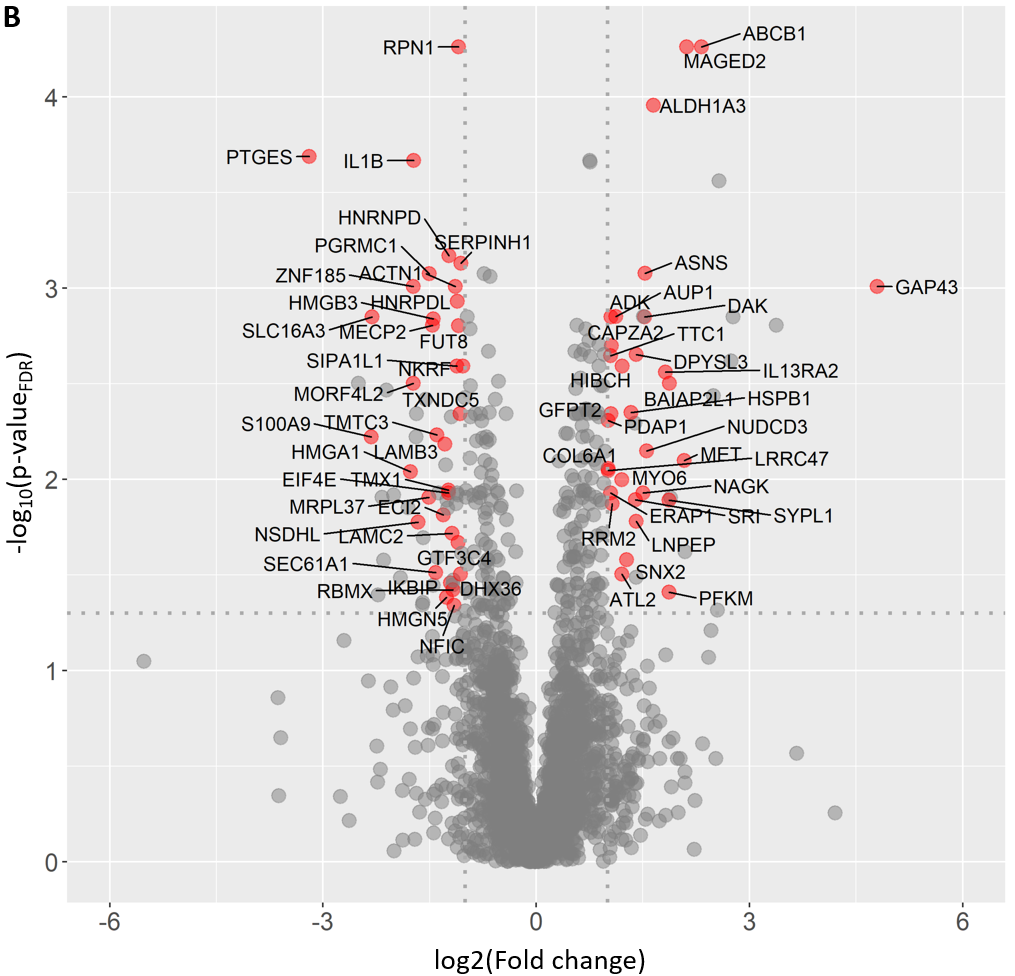


**Supplementary figure 2. Volcano plot visualization of detected proteins (as dots) by LC-MS/MS analysis. (A) DU145 vs. DU145-DR and (B) PC3 vs. PC3-DR cell lines.** Significantly changed (FDR-corrected p-value ≤ 0.05) proteins are shown above the horizontal dashed line. At least two-fold down-regulated proteins are presented left from the left vertical line, while more than two-fold up-regulated proteins are shown right from the right vertical dashed lines. Upper right quarter contains the up-regulated, while the left one shows the down-regulated proteins in the DOC-resistant DU145-DR (A) and PC3-DR (B) cells.
