# Supplementary material for: Comparative proteome analysis identified CD44 as a possible serum marker for docetaxel resistance in castration‐resistant prostate cancer
Source: J Cell Mol Med. 2021 Dec 30;26(4):1332–7. doi: 10.1111/jcmm.17141 (PMC8831956; doi:10.1111/jcmm.17141)
Supplement: Supplementary file 3 — Fig S3 [file JCMM-26-1332-s007.docx]

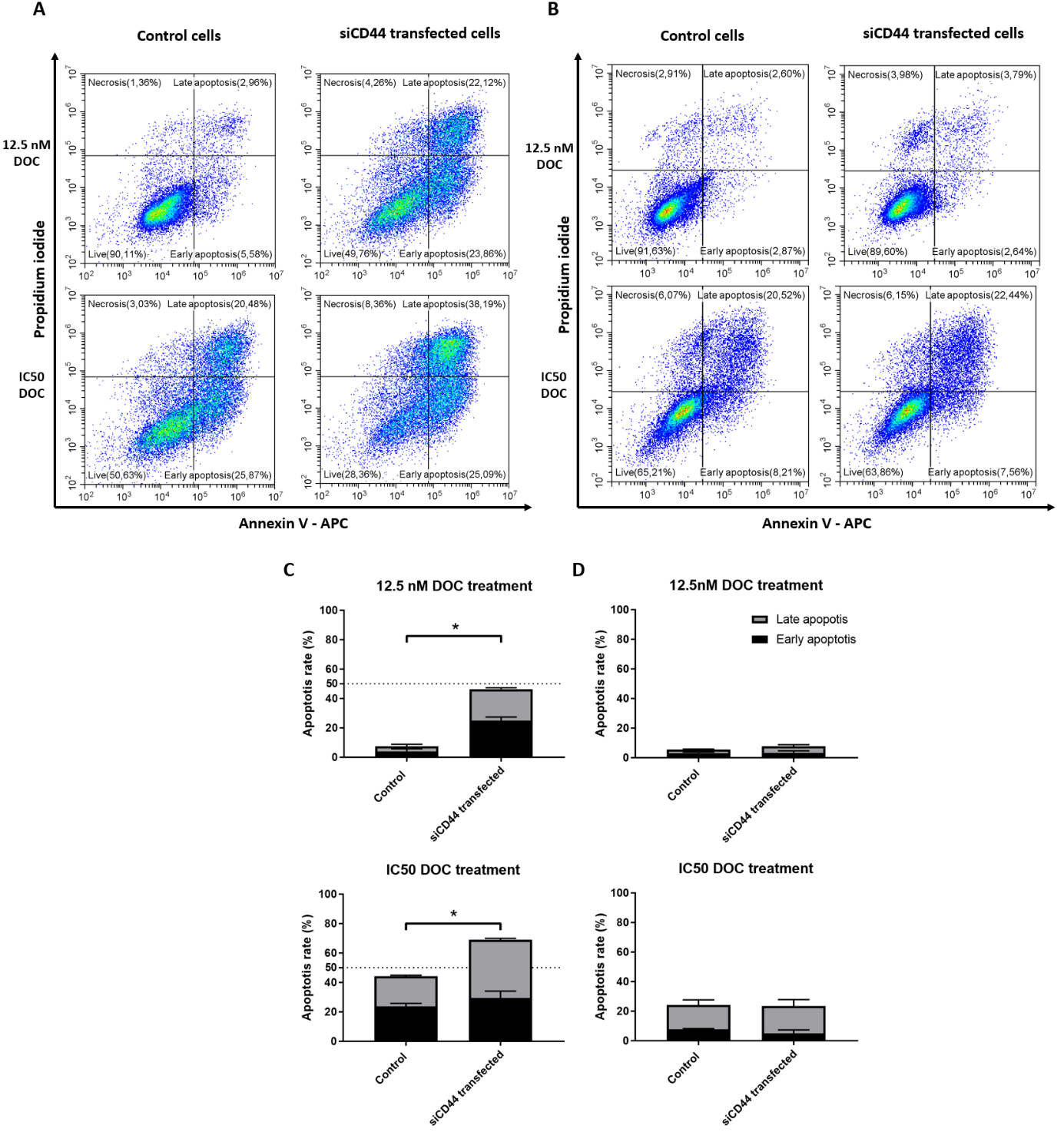


**Supplementary figure 3. Apoptosis analysis by flow cytometry was performed on both DOC resistant cell lines.** Regarding the experiments done on DU145-DR cells (A and C) we could detect significant changes (*p<0.01). The mean rate of the living DU145-DR cells due to siCD44 transfection changed from 88.59% to 47.57 % (under 12.5 nM DOC treatment), and from 50.07% to 27.22% (IC50 = 100 nM DOC). Meanwhile the mean rate of the apoptotic cells in the population raised from 7.50% to 46.30% (12.5 nM DOC), and from 44.11% to 65.92% (IC50 = 100 nM DOC). The same experiments with PC3-DR cells did not show similar changes (B and D; IC50 DOC concentration for PC3-DR cells: 200 nM).
