# Supplementary material for: Comparative proteome analysis identified CD44 as a possible serum marker for docetaxel resistance in castration‐resistant prostate cancer
Source: J Cell Mol Med. 2021 Dec 30;26(4):1332–7. doi: 10.1111/jcmm.17141 (PMC8831956; doi:10.1111/jcmm.17141)
Supplement: Supplementary file 4 — Table S1 [file JCMM-26-1332-s008.docx]

| **PC3-DR compared to PC3** | | | | |  | **DU145-DR compared to DU145** | | | | |
| --- | --- | --- | --- | --- | --- | --- | --- | --- | --- | --- |
| **Gene abbreviation** | **Uniprot ID** | **Protein name** | **Fold change** | **FDR-corrected p-value** |  | **Gene abbreviation** | **Uniprot ID** | **Protein name** | **Fold change** | **FDR-corrected p-value** |
| **GAP43** | P17677 | Neuromodulin | 27.77 | 0.0009785 |  | **ABCB1** | P08183 | Multidrug resistance protein 1 | 17.23 | 0.0000122 |
| **ABCB1** | P08183 | Multidrug resistance protein 1 | 5.00 | 0.0000546 |  | **C19orf21** | Q8IVT2 | Uncharacterized protein C19orf21 | 8.44 | 0.0010750 |
| **MAGED2** | Q9UNF1 | Melanoma-associated antigen D | 4.34 | 0.0000546 |  | **LIMCH1** | Q9UPQ0 | LIM and calponin homology domains-containing protein 1 | 8.16 | 0.0028886 |
| **MET** | P08581 | Hepatocyte growth factor receptor | 4.24 | 0.0079544 |  | **LMO7** | Q8WWI1 | LIM domain only protein 7 | 7.683 | 0.0000032 |
| **BAIAP2L1** | Q9UHR4 | Brain-specific angiogenesis inhibitor 1-associated protein 2-like protein 1 | 3.67 | 0.0031445 |  | **CALD1** | Q05682 | Caldesmon | 7.60 | 0.0003924 |
| **SYPL1** | Q16563 | Synaptophysin-like protein 1 | 3.66 | 0.0128254 |  | **PDLIM5** | Q96HC4 | PDZ and LIM domain protein 5 | 7.13 | 0.0038140 |
| **PFKM** | P08237 | 6-phosphofructokinase (muscle type) | 3.65 | 0.0388029 |  | **SDPR** | O95810 | Serum deprivation-response protein | 7.01 | 0.0000001 |
| **IL13RA2** | Q14627 | Interleukin-13 receptor subunit alpha-2 | 3.52 | 0.0027476 |  | **TAOK3** | Q9H2K8 | Serine/threonine-protein kinase TAO3 | 6.60 | 0.0005525 |
| **ALDH1A3** | P47895 | Aldehyde dehydrogenase family 1 member A3 | 3.13 | 0.0001106 |  | **MVP** | Q14764 | Major vault protein | 6.11 | 0.0000014 |
| **NUDCD3** | Q8IVD9 | NudC domain-containing protein 3 | 2.93 | 0.0070859 |  | **EHD2** | Q9NZN4 | EH domain-containing protein 2 | 5.93 | 0.0000175 |

**Supplementary table 1.** **Top 10 upregulated proteins (ranked by fold change) between the two DOC-sensitive and DOC-resistant PC cell lines as measured by LC-MS/MS analysis.** FDR – false discovery rate.
