# Supplementary material for: Comparative proteome analysis identified CD44 as a possible serum marker for docetaxel resistance in castration‐resistant prostate cancer
Source: J Cell Mol Med. 2021 Dec 30;26(4):1332–7. doi: 10.1111/jcmm.17141 (PMC8831956; doi:10.1111/jcmm.17141)
Supplement: Supplementary file 5 — Table S2 [file JCMM-26-1332-s001.docx]

|  | n | **MET**  serum cc. (ng/ml) | p | **CD44** serum cc. (pg/ml) | p | All patients | **LNPEP** negative | | **LNPEP** positive | | p | **PSA** serum cc. (ng/ml) | p |
| --- | --- | --- | --- | --- | --- | --- | --- | --- | --- | --- | --- | --- | --- |
|  |  |  |  |  |  |  |  |  |  |  |  |  |  |
|  |  | median (range) |  | median (range) |  | n (%) | n | % | n | % |  | median (range) |  |
| **Age (years)** |  |  |  |  |  |  |  |  |  |  |  |  |  |
| < 71 | 33 | 341.50 (194.90-725.60) | 0.273 | 851.80 (78.00-5000.00) | 0.964 | 26 | 19 | 73.1 | 7 | 26.9 | 0.827 | 70.10 (2.55-1638.25) | 0.611 |
| ≥ 71 | 33 | 354.80 (149.70-1058.80) |  | 922.30 (78.00-5000.00) |  | 27 | 19 | 70.4 | 8 | 29.6 |  | 88.79 (4.20-7312.04) |  |
| **Primary therapy** |  |  |  |  |  |  |  |  |  |  |  |  |  |
| no | 47 | 341.50 (175.40-1058.80) | 0.944 | 922.30 (78.00-3910.00) | 0.350 | 38 | 26 | 68.4 | 12 | 31.6 | 0.399 | 108.68 (4.20-7312.04) | 0.125 |
| yes | 19 | 354.80 (149.70-552.00) |  | 714.70 (78.00-5000.00) |  | 15 | 12 | 80.0 | 3 | 20.0 |  | 74.31 (2.55-347.38) |  |
| **Primary RPE** |  |  |  |  |  |  |  |  |  |  |  |  |  |
| no | 54 | 345.90 (149.70-1058.80) | 0.861 | 897.95 (78.00-5000.00) | 0.454 | 43 | 30 | 69.8 | 13 | 30.2 | 0.518 | 88.22 (4.20-7312.04) | 0.267 |
| yes | 12 | 353.35 (219.80-552.00) |  | 748.75 (78.00-5000.00) |  | 10 | 8 | 80.0 | 2 | 20.0 |  | 66.68 (2.55-347.38) |  |
| **Primary RAD** |  |  |  |  |  |  |  |  |  |  |  |  |  |
| no | 59 | 341.50 (175.40-1058.80) | 0.747 | 917.80 (78.00-5000.00) | 0.662 | 48 | 34 | 70.8 | 14 | 29.2 | 0.665 | 88.79 (2.55-7312.04) | 0.396 |
| yes | 7 | 354.80 (149.70-507.60) |  | 714.70 (180.10-5000.00) |  | 5 | 4 | 80.0 | 1 | 20.0 |  | 74.31 (5.09-273.29) |  |
| **ECOG PS** |  |  |  |  |  |  |  |  |  |  |  |  |  |
| 0 | 60 | 333.85 (149.70-839.60) | 0.719 | 897.95 (78.00-5000.00) | 0.221 | 49 | 34 | 69.4 | 15 | 30.6 | 0.256 | 87.32 (2.55-7312.04) | 0.481 |
| 1-2 | 5 | 363.90 (175.40-1058.80) |  | 839.3 (78.00-2383.00) |  | 3 | 3 | 100.0 | 0 | 0.0 |  | 209.64 (7.89-1037.48) |  |
| unknown | 1 |  |  |  |  | 1 |  |  |  |  |  |  |  |
| **Lymph node status** |  |  |  |  |  |  |  |  |  |  |  |  |  |
| N - | 37 | 321.60 (149.70-1058.80) | 0.458 | 938.4 (78.00-5000.00) | 0.256 | 32 | 24 | 75.0 | 8 | 25.0 | 0.510 | 120.32 (5.09-7312.04) | 0.080 |
| N + | 29 | 369.40 (188.50-764.80) |  | 786.60 (180.00-5000.00) |  | 21 | 14 | 66.7 | 7 | 33.3 |  | 51.59 (2.55-939.14) |  |
| unknown | 0 |  |  |  |  |  |  |  |  |  |  |  |  |
| **Visceral mets.** |  |  |  |  |  |  |  |  |  |  |  |  |  |
| no | 52 | 321.60 (149.70-1058.80) | 0.442 | 938.4 (78.00-5000.00) | 0.117 | 43 | 30 | 69.8 | 13 | 30.2 | 0.518 | 82.745 (2.55-7312.04) | 0.919 |
| yes | 14 | 369.40 (188.50-764.80) |  | 786.6 (180.10-5000.00) |  | 10 | 8 | 80.0 | 2 | 20.0 |  | 92.58 (5.09-625.71) |  |
| unknown | 0 |  |  |  |  |  |  |  |  |  |  |  |  |
| **Bone mets.** |  |  |  |  |  |  |  |  |  |  |  |  |  |
| no | 2 | 247.60 (232.40-262.80) | 0.103 | 1472.40 (917.80-2027.00) | 0.373 | 2 | 1 | 50.0 | 1 | 50.0 | 0.487 | 57.79 (26.23-89.35) | 0.496 |
| yes | 64 | 352.55 (149.70-1058.80) |  | 859.55 (78.00-5000.00) |  | 51 | 37 | 72.6 | 14 | 27.4 |  | 87.81 (2.55-7312.04) |  |
| unknown | 0 |  |  |  |  |  |  |  |  |  |  |  |  |
| **PSA response** |  |  |  |  |  |  |  |  |  |  |  |  |  |
| response | 46 | 366.65 (175.40-1058.80) | 0.374 | 806.80 (78.00-5000.00) | 0.664 | 36 | 25 | 65.8 | 11 | 29.0 | 0.512 | 87.32 (2.55-4224.24) | 0.957 |
| no response | 11 | 296.60 (219.80-552.00) |  | 917.80 (78.00-3910.00) |  | 10 | 8 | 80.0 | 2 | 20.0 |  | 87.81 (5.09-1638.25) |  |
| unknown | 9 |  |  |  |  | 7 |  |  |  |  |  |  |  |
| **PSA response** |  |  |  |  |  |  |  |  |  |  |  |  |  |
| > 30% | 36 | 366.65 (175.40-1058.80) | 0.785 | 819.20 (78.00-5000.00) | 0.904 | 27 | 17 | 63.0 | 10 | 37.0 | 0.095 | 82.75 (2.55-1037.48) | 0.533 |
| < 30% | 22 | 324.30 (194.90-839.60) |  | 853.30 (78.00-3910.00) |  | 20 | 17 | 85.0 | 3 | 15.0 |  | 116.53 (5.09-4224.24) |  |
| unknown | 8 |  |  |  |  | 6 |  |  |  |  |  |  |  |
| **PSA response** |  |  |  |  |  |  |  |  |  |  |  |  |  |
| > 50% | 31 | 369.40 (175.40-1058.80) | 0.668 | 709.50 (78.00-5000.00) | 0.236 | 23 | 15 | 65.2 | 8 | 34.8 | 0.285 | 70.10 (2.55-1037.48) | 0.225 |
| < 50% | 27 | 321.60 (194.90-839.60) |  | 917.80 (78.00-3910.00) |  | 24 | 19 | 79.2 | 5 | 20.8 |  | 102.94 (5.09-4224.24) |  |
| unknown | 8 |  |  |  |  | 6 |  |  |  |  |  |  |  |
| **PSA response** |  |  |  |  |  |  |  |  |  |  |  |  |  |
| > 90% | 16 | 350.60 (252.00-725.60) | 0.903 | 660.55 (180.10-5000.00) | 0.230 | 37 | 27 | 73.0 | 10 | 27.0 | 0.852 | 68.16 (2.55-399.63) | 0.417 |
| < 90% | 42 | 340.90 (175.40-1058.80) |  | 897.95 (78-3910) |  | 10 | 7 | 70.0 | 3 | 30.0 |  | 88.22 (4.20-4224.24) |  |
| unknown | 8 |  |  |  |  | 6 |  |  |  |  |  |  |  |
| **Radiographic prg.** |  |  |  |  |  |  |  |  |  |  |  |  |  |
| no | 18 | 342.75 (188.50-839.60) | 0.790 | 622.65 (180.10-3393.00) | 0.162 | 15 | 10 | 66.7 | 5 | 33.3 | 0.692 | 78.17 (4.20-837.05) | 0.533 |
| yes | 24 | 340.90 (175.40-750.00) |  | 872.70 (78.00-5000.00) |  | 22 | 16 | 72.7 | 6 | 27.3 |  | 88.22 (5.09-1638.25) |  |
| unknown | 24 |  |  |  |  | 16 |  |  |  |  |  |  |  |
|  |  |  |  |  |  |  |  |  |  |  |  |  |  |
| **Whole cohort** | 66 | 345.90 (149.70-1058.80) |  | 872.70 (78.00-5000.00) |  | 53 | 38 | 71.7 | 15 | 28.3 |  | 87.81 (2.55-7312.04) |  |

**Supplementary table 2. Descriptive statistical evaluation of baseline MET, CD44 and PSA levels and dichotomized LNPEP values with patients’ clinicopathological data.** ECOG PS – Eastern Cooperative Oncology Group Performance Status, RPE – radical prostatectomy, RAD – irradiation, mets – metastasis, prg - progression.
