# Supplementary material for: Comparative proteome analysis identified CD44 as a possible serum marker for docetaxel resistance in castration‐resistant prostate cancer
Source: J Cell Mol Med. 2021 Dec 30;26(4):1332–7. doi: 10.1111/jcmm.17141 (PMC8831956; doi:10.1111/jcmm.17141)
Supplement: Supplementary file 6 — Table S3 [file JCMM-26-1332-s005.docx]

| **Variables** |  | **Overall survival** | | |
| --- | --- | --- | --- | --- |
|  |  | **HR** | **95% CI** | **p** |
| Age | < 71 | ref. |  |  |
|  | ≥ 71 | 1.456 | 0.851 - 2.490 | 0.170 |
| ECOG PS | 0 | ref. |  |  |
|  | 1-2 | 1.774 | 1.029 - 3.059 | **0.039** |
| Visceral mets. | no | ref. |  |  |
|  | yes | 1.351 | 0.720 - 2.537 | 0.349 |
| Lymph node mets. | no | ref. |  |  |
|  | yes | 1.024 | 0.596 - 1.759 | 0.933 |
| Bone mets. | no | ref. |  |  |
|  | yes | 0.678 | 0.164 - 2.804 | 0.592 |
| Primary RPE | no | ref. |  |  |
|  | yes | 1.181 | 0.606 - 2.301 | 0.624 |
| Primary RAD | no | ref. |  |  |
|  | yes | 1.331 | 0.566 - 3.132 | 0.512 |
| PSA baseline (median) | < 88 ng/ml | ref. |  |  |
|  | > 88 ng/ml | 1.506 | 0.866 - 2.619 | 0.146 |
| PSA response | no response | ref. |  |  |
|  | response | 0.291 | 0.141 - 0.602 | **0.001** |
| PSA response | < 30% | ref. |  |  |
|  | > 30% | 0.455 | 0.250 - 0.828 | **0.001** |
| PSA response | < 50% | ref. |  |  |
|  | > 50% | 0.45 | 0.249 - 0.814 | **0.008** |
| PSA response | < 90% | ref. |  |  |
|  | > 90% | 0.634 | 0.327 - 1.227 | 0.176 |
| CD44 (median) | < 872.7 pg/ml | ref. |  |  |
|  | > 872.7 pg/ml | 1.976 | 1.150 - 3.395 | **0.014** |
| CD44 (upper 25%) | < 1237 pg/ml | ref. |  |  |
|  | > 1237 pg/ml | 1.313 | 1.069 - 1.612 | **0.009** |
| MET (median) | < 345.9 ng/ml | ref. |  |  |
|  | > 345.9 ng/ml | 1.119 | 0.655 - 1.913 | 0.680 |
| LNPEP | negative | ref. |  |  |
|  | positive | 0.837 | 0.429 - 1.634 | 0.602 |

**Supplementary table 3.** **Univariable Cox survival analysis.** Patients’ clinical parameters and marker levels were correlated with OS. ECOG PS (1-2), PSA response and the median and upper 25% of pre-treatment CD44 values were found to be significantly correlated with poor OS. Significant p-values are indicated in bold (p≤0.05). ECOG PS – Eastern Cooperative Oncology Group Performance Status, mets – metastasis, RPE – radical prostatectomy, RAD – irradiation.
