# Supplementary material for: Comparative proteome analysis identified CD44 as a possible serum marker for docetaxel resistance in castration‐resistant prostate cancer
Source: J Cell Mol Med. 2021 Dec 30;26(4):1332–7. doi: 10.1111/jcmm.17141 (PMC8831956; doi:10.1111/jcmm.17141)
Supplement: Supplementary file 7 — Table S4 [file JCMM-26-1332-s003.docx]

|  | **Overall survival** | | |
| --- | --- | --- | --- |
|  | **HR** | **95% CI** | **p** |
| **Baseline model** |  |  |  |
| ECOG PS (0 vs.1- 2) | 2.107 | 0.977 - 4.545 | 0.057 |
| Baseline PSA (median) >88 ng/ml | 1.000 | 0.464 - 2.155 | 0.999 |
| CD44 (>1237 pg/ml) | 1.304 | 1.051 - 1.618 | **0.016** |
|  |  |  |  |
| **Follow-up model** |  |  |  |
| ECOG PS (0 vs.1- 2) | 1.519 | 0.817 - 2.825 | 0.187 |
| Any PSA decline (yes) | 0.227 | 0.103 - 0.502 | **<0.001** |
| CD44 (>1237 pg/ml) | 1.531 | 1.193 - 1.964 | **0.001** |

**Supplementary table 4.** **Multivariable survival analysis.** The baseline model included parameters available before DOC treatment start while the follow-up model included also parameters, such as PSA response, that are only available after treatments start. Higher CD44 serum levels (>1237 pg/ml) in both multivariable models, and PSA decline in our follow-up model proved to be significant, revealing these parameters as independent predictors of poor OS. Significant p-values are indicated in bold (p≤0.05).
