# Supplementary material for: Comparative proteome analysis identified CD44 as a possible serum marker for docetaxel resistance in castration‐resistant prostate cancer
Source: J Cell Mol Med. 2021 Dec 30;26(4):1332–7. doi: 10.1111/jcmm.17141 (PMC8831956; doi:10.1111/jcmm.17141)
Supplement: Supplementary file 8 — Supplementary Material [file JCMM-26-1332-s004.docx]

# **Supplementary Methods**

# **Detailed description of LC-MS/MS analysis**

## Sample Preparation of Cells for Label-Free LC-MS/MS

Parental PC cell lines and their resistant subclones were harvested at about 70% confluence. The cell pellets were lysed using 0.1% NaDOC in TBS supplemented with protease inhibitors (complete mini, Roche, Penzberg, Germany) and Benzonase (25 U per sample, Merck). Samples were sonicated on ice for 10 min and lysis buffer (30 mM Tris, 7M urea, 2 M thiourea, 0.1% SDS, pH 8.5) was added for protein solubilization. Samples were centrifuged (10 min; 16100 x g) and supernatant concentrations were determined using the Bradford assay (Bio-Rad, Hercules, CA). 30 µg of proteins were applied to 18% Tris-Glycine-Gels (Anamed Elektrophorese, Rodau, Germany) and run into the gel at 100 V for 15 min to concentrate the proteins in single bands which were stained with Coomassie and cut from the gels. Digestion with trypsin (SERVA, Heidelberg, Germany) was performed in 10 mM ammonium bicarbonate buffer overnight at 37 °C. Peptides were extracted, dried in a vacuum centrifuge and dissolved in 0.1% TFA. The peptide concentrations were determined via amino acid analysis as described previously (Megger et al., Mol Cell Proteomics. 2013 Jul;12(7):2006-20.).

## LC-MS/MS Parameters

LC–MS/MS analysis was performed as described before (Megger et al., Mol Cell Proteomics. 2013 Jul;12(7):2006-20.). Briefly, 300 ng tryptic peptides were applied to an Ultimate 3000 RSLCnano HPLC coupled to an Orbitrap Elite instrument (both Thermo Sientific, Bremen, Germany). Peptides were concentrated on a C18 trap column (Acclaim PepMap 100; 100 μm × 2 cm, 5 μm, 100 Å) within 7 min at a flow rate of 30 μl/min with 0.1% TFA and then transferred to a Nano Viper C18 analytical column (Acclaim PepMap RSLC; 75 μm × 50 cm, 2 μm, 100 Å). Peptides were separated with a gradient from 5%–40% solvent B over 98 min at 400 nl/min and 60°C (solvent A: 0.1% FA; solvent B: 0.1% FA, 84% ACN). Full-scan mass spectra were acquired in profile mode at a resolution of 60,000 at 400 m/z within a mass range of 350–2000 m/z. MS/MS spectra were acquired at a resolution of 5,400. For MS/MS measurements, the 20 most abundant peptide ions were fragmented by collision-induced dissociation (CID, NCE 35).

## Protein Identification and Quantification

Proteins identification was conducted with Proteome Discoverer v.1.4 (Thermo Fisher Scientific). Spectra were searched against the UniProtKB/Swiss-Prot database (Release 2016_05; 70625 entries) using Mascot v.2.5 (Matrix Science, London, UK). Taxonomy setting was Homo sapiens and mass tolerances were 5 ppm and 0.4 Da for precursor and fragment ions, respectively. Dynamic and static modifications were considered for methionine (oxidation) and cysteine (carbamidomethyl), respectively. The false discovery rate (FDR) was calculated with the Target Decoy PSM Validator and identifications with an FDR > 1% were rejected. Progenesis QI v.2.0.5387.52102 (Nonlinear Dynamics, Durham, NC, USA) was used for label-free quantification. Raw files were aligned to a reference run and a master map of features was applied to all experimental runs to adjust for differences in retention time. Ion charge states of 2+, 3+, and 4+ with a minimum of three isotope peaks were considered. Statistical analysis was conducted using R and t-tests were calculated using arcsinh-transformed normalized protein abundances. Ratios of mean abundances (RoM) were determined based on non-transformed data. P-values were FDR-adjusted according to Benjamini and Hochberg and proteins quantified with minimum 2 unique peptides, a pFDR-value ≤ 0.05 and an absolute RoM ≥ 2 were considered as significantly differentially abundant.

# **Biomarker selection**

We established a workflow for the prediction of potentially secreted proteins as these may be found in patients’ serum samples. Our workflow included existing prediction programs (SignalP 4.1, SecretomeP 2.0, TargetP 1.1, TMHMM 2.0) and databases (Uniprot, Human Protein Atlas, NCBI, ExoCarta). In order to install our workflow, we performed optimized the method in a set of 200 known-to-be secreted and 200 non-secreted proteins. This was then used to develop improved secreted peptide prediction based on our binary weighted table. Finally, an optimized cut-off value determined to obtain maximal sensitivity and specificity on the test data set. This method then was applied to our identified LC-MS/MS-based dataset.

## **Validation of selected markers at the mRNA and protein levels in cell lines**

## RNA isolation and qRT-PCR

Total RNA was isolated from cultured cells using the RNeasy Plus Mini Kit (Qiagen, Hilden, Germany) according to manufacturer’s recommendations. RNA concentrations were measured by using a Qubit 4 Fluorometer (Invitrogen, Carlsbad, CA, USA,) with the Qubit RNA BR Assay Kit (Invitrogen). RNA (1 µg) was transcribed to cDNA using High-Capacity cDNA Reverse Transcription Kit (Thermo Fisher Scientific, Waltham, MA, USA. Real-time qPCR was performed in duplicate in a final reaction volume of 20 µl using the StepOne Plus Real-Time PCR System (Applied Biosystems, Foster City, CA, USA). We used the TaqMan Gene Expression Assay for CD44 (Hs01075864_m1) and the HPRT1 gene was used as a housekeeping gene (Hs02800695_m1). Reaction plate was initially hold on 50°C for 2 min, then polymerase was activated with an incubation of 10 min at 95°C. cDNA samples were denatured at 95°C for 15 sec and extended at 60°C for 1 min for with a total 40 cycles. For data evaluation the fold change of the target genes were calculated by the ∆∆Ct method.

## Western Blot analysis

Twenty μg protein per sample was separated on bolt 4-12% Bis-Tris Plus Gels (Invitrogen) and blotted onto nitrocellulose membrane of iBlot 2 Transfer Stacks using iBlot 2 Gel Transfer Device. After blocking in 5% NFDM/TBST membrane were incubated with anti-CD44 rabbit polyclonal antibody (1:3000, Abcam, Cambridge, ab157107, Immunogen: NP_000601.3) overnight at 4°C which was followed by thorough washing and incubation with goat anti-rabbit (1:6000, Abcam, Cat. No: ab6721) secondary antibody for 1h at room temperature. For loading control anti-GAPDH mouse monoclonal antibody was applied (1:8000; Abcam, ab8245) with goat anti-mouse secondary antibody (1:6000, Abcam, ab6789). After three washes of TBST buffer, bound proteins were detected using SuperSignal West Pico Plus Chemiluminescent Substrate (Thermo Fisher Scientific) and captured by iBright FL1000 Imaging System (Invitrogen). The densitometry analyses for quantifying protein expression changes we used ImageJ software.

# **Functional experiments**

## CD44 gene silencing

SMARTpool on-target plus siRNA against CD44 (Dharmacon, Lafayette, CO, USA, Cat. No: L-009999-00-0005) and non-targeting control pool on-target plus siRNA (Dharmacon, D-0018101005) reached 10 μM concentration after dissolving in 1x siRNA Buffer (Dharmacon, B-002000UB100). We used Lipofectamine RNAiMAX Transfection Reagent (Invitrogen,13778150) as transfection agent and Opti-MEM Reduced Serum Medium (Gibco) for siRNA-lipid complex according to manufacturer’s instructions.

First, 150.000 cells per well were seeded in 6-well plates in 2 mL medium supplemented with 10% FBS. Each cell line was transfected with 40 pmol siRNA. Non-targeting control pool siRNA was applied as negative control. Medium was changed 8h after transfection. Gene silencing was monitored by Western blot and ELISA.

## Flow cytometry

Forty-eight hours after gene silencing the cells were treated for 72h with the maintenance dose (12.5 nM) and the IC50 doses of DOC. At the end of the incubation time subconfluent cells were harvested and pelleted with their supernatants for apoptosis analysis. Then, samples were washed twice with cold Cell Staining Buffer (BioLegend, San Diego, CA, USA). Cell samples were diluted in Annexin V Binding Buffer at the concentration of 0.25 – 1.0 x 107 cells/ml. 2.5µl APC Annexin V and 5µl propidium iodide (PI) solution were added to 100µl cell suspension (APC Annexin V Apoptosis Detection Kit with PI, BioLegend). Samples were incubated at room temperature in dark. Just before flow cytometry measurements 200 µl annexin V binding buffer was added to the samples. Samples were vortexed just before flow cytometry analyses which were performed on CytoFLEX Flow Cytometer and the settings were done by the CytExpert 2.3 software (Beckman Coulter, IN, USA).

## Statistical analysis

Regarding ELISA evaluations for paired comparisons between groups, the nonparametric, 2-sided Wilcoxon rank-sum test was applied. Survival analyses were done using Kaplan–Meier curves, log-rank test and univariable Cox proportional hazards regression analysis. For multivariable analysis, Cox regression models were used. Variables with effect on survival in univariable analysis (p≤0.05) were considered in the Cox proportional hazards regression models. Statistical tests were performed using SPSS 23.0 software (IBM, Chicago, IL, USA).

Flow cytometry apoptosis analysis was evaluated by GraphPad Prism 8.0.1. software (GraphPad Software, San Diego, CA, USA). For these statistical analyses (p≤0.01) multiple t-test was applied.
