# Supplementary material for: Comparative proteome analysis identified CD44 as a possible serum marker for docetaxel resistance in castration‐resistant prostate cancer
Source: J Cell Mol Med. 2021 Dec 30;26(4):1332–7. doi: 10.1111/jcmm.17141 (PMC8831956; doi:10.1111/jcmm.17141)
Supplement: Supplementary file 9 — Supplementary Material [file JCMM-26-1332-s002.doc]

# Supplementary Discussion

ABCB1 also known as MDR1 is a well-known drug transport protein, which is involved in chemotherapy-resistance of tumor cells by pumping drugs out of the tumor cells. Zhu et al. showed that acquired DOC-resistance of PC cell lines can be overcome by the inhibition of ABCB1 ^1^. In a subsequent study using *in vitro* and *in vivo* techniques they showed that anti-androgen treatments with bicalutamide and enzalutamide are able to inhibit the efflux activity of ABCB1 ^2^. In accordance, our present proteome analysis found ABCB1 as one of the most strongly upregulated protein in both analyzed resistant cell PC cell lines.

In addition, we found two further proteins, SYPL1 (Synaptophysin-like protein 1) and HSPB1 (Heat shock protein beta 1, also known as Hsp27) to be consequently upregulated in both of our DOC-resistant cell lines. Since these were not identified as potentially secreted proteins by our prediction method, they were not subjected to further investigations in this study. The role of SYPL1 in cancer is poorly known so far, although it has been suggested as a potential prognostic biomarker in hepatocellular carcinoma (HCC) and its expression has found to be associated with epithelial-to-mesenchymal transition (EMT) in HCC ^3^.

HSPB1 is an intracellular molecular chaperone involved in stress response. HSPs are frequently overexpressed in various tumors and have been shown to help cells escaping apoptosis ^4^. Foster et al. demonstrated that HSPB1 is able to independently predict poor clinical outcome in PC at the time of diagnosis ^5^. It has been also described that Hsp27 can interact with androgen receptor (AR) leading gene transcription of AR-regulated genes ^6^. HSPs have formerly been considered as a therapy target but could not be established as a treatment option for PC so far ^7, 8^.

Our proteomics analysis revealed GAP43 (Growth-associated protein 43, also known as Neuromodulin) as the most strongly upregulated protein (27.8-fold) in PC3-DR cells. GAP43 is a neuronal protein and has a physiological role in nerve growth. It has been demonstrated that GAP43 is overexpressed in various brain tumor cell lines and a tumor promoting capability has been related to GAP43 in glioblastomas as well ^9^. Additionally, Zhang et al. have shown that GAP43 might be used as a biomarker in non-small cell lung cancer for the prediction of brain metastases ^10^. However, its expression and role in PC has not been evaluated yet.

We compared our results to three formerly published studies performed by high throughput hypothesis-free screening for the identification of protein or mRNA markers of DOC-resistance in PC cell lines.

In the first study, Zhao *et al.* compared PC3 cell line with their DOC-resistant sub-line (PC3-Rx) at the protein level by using iTRAQ-mass spectrometry and identified 427 upregulated proteins in PC3-Rx cells. They selected secreted proteins for further analyses and found MIC-1 and AGR2 as potential biomarkers and drug targets in DOC-resistance mCRPC ^11^. These proteins were not identified in our proteomics analyses.

In the second study, O’Connell *et al.* performed a proteomic comparison of three DOC-sensitive (PC3, DU145 and 22RV1) and-resistant cell lines. They selected proteins with a significant fold-change at least two of the resistant cell lines and assigned them to respective pathways in order to reveal their functions, interactions and regulations and found that heat shock response proteins and EMT-related markers were deregulated in DOC-resistant PC cells ^12^.

In the third study, Marín-Aguilera *et al.* used whole genome arrays for comparative global gene expression analysis between PC3 and DU145 vs. PC3R (DOC-resistant) and DU145R cells. According to their analysis 243 genes were significantly upregulated in both DOC-resistant cell lines. After selection steps 18 genes were validated in cell lines and were further tested in mCRPC tumor tissue samples by RT-qPCR. They could confirm AREG, CDH1, IFIH1 and EPCAM to be downregulated in tumor samples of DOC-resistant patients ^13^.

We cross referenced differently expressed proteins identified in our analyses with those found in the above three studies and found ANXA3, ANXA6, SND1, FLNC, NAMPT, ACTN1 and PYGB as consequently upregulated and PDIA3, OAT, LDHA, FLNB and CKAP4 as consequently downregulated proteins in at least two independent studies (supplementary figure 1.). None of these proteins were identified as secreted ones according to our above mentioned bioinformatics selection method.

Based on our LC-MS/MS analysis and bioinformatics filtering, we selected CD44, MET, LNPEP, GSN and IL13RA2 for serum analysis. We did not find any correlation between serum levels of MET, LNPEP, GSN and IL13RA2 and clinicopathological or survival data of mCRPC patients who underwent DOC therapy. On the other hand, elevated serum CD44 levels were significantly associated with poor overall survival. This correlation remained significant also in the multivariable analysis revealing CD44 as an independent predictor of poor survival in patients who underwent DOC-therapy. However, our results suggest no value for repeat serum CD44 analysis in therapy monitoring of DOC as we found no significant changes in their levels during DOC treatment.

To explore CD44’s role in DOC-resistance functional *in vitro* experiments were performed, which revealed that siRNA-mediated CD44 silenced DU145-DR cells showed higher rate of apoptotic cells to DOC treatment compared to non-transfected cells. This suggests the functional involvement of CD44 in DOC-resistance. Therefore, CD44 might be considered as a therapeutic target to overcome DOC-resistance in PC. CD44 levels were only differently abundant and functionally relevant in the DU145 cell lines but not in PC3. This underlines the observation, that resistance mechanisms are heterogenous and more than one mechanism may be involved in DOC resistance.

CD44 is a transmembrane glycoprotein involved in several cellular processes such as cell adhesion, differentiation, proliferation, migration and survival. These functions are important for the normal cells under physiological conditions, Moreover, different isoforms or altered expression of CD44 has been associated with certain malignancies ^14, 15^. CD44 is considered as a cancer stem cell (CSC) marker in several tumor entities. CSCs are not in replicative cellular state and therefore they are able to avoid the cytotoxic effect of chemotherapies. In order to configure themselves into quiescence status CSCs’ interactions with their microenvironment are remarkably important. Since the extracellular domain of CD44 interacts with numerous surrounding molecules such as growth factors or components of the extracellular matrix, it has been suggested to be an important regulator of cancer stemness. Furthermore, the plasticity of CSCs enables tumorigenesis, self-renewal, cellular transformation (e.g. EMT) or metastasis formation ^16-19^. Soluble serum CD44 was shown to arise by proteolytic cleavage of its ectodomain by various membrane-associated matrix metalloproteinases ^20^. Kato *et al.* have investigated the CD44v8-10 splice variant levels in serum exosomes of healthy controls, DOC-naive and DOC-resistant PC patients. They found higher levels of CD44v8-10 in DOC-resistant patients’ samples ^21^. Lai *et al.* performed *in vitro* experiments on DOC-sensitive and resistant cell lines, focusing on the migration and invasion abilities of DOC-resistant cells. Their FACS analysis revealed that the CD44+ cells are overrepresented in the resistant population which were interpreted as cancer stem cell (CSC)-like cells. Moreover, they could show reduced migratory and invasive capabilities in CD44 silenced cells ^22^. Overall, these data are in line with our findings, suggesting CD44 to be involved in DOC resistance in mCRPC.

References

1. Zhu Y, Liu C, Nadiminty N, et al. Inhibition of ABCB1 expression overcomes acquired docetaxel resistance in prostate cancer. *Mol Cancer Ther*. 2013;12(9):1829-36.

2. Zhu Y, Liu C, Armstrong C, Lou W, Sandher A, Gao AC. Antiandrogens Inhibit ABCB1 Efflux and ATPase Activity and Reverse Docetaxel Resistance in Advanced Prostate Cancer. *Clin Cancer Res*. 2015;21(18):4133-42.

3. Chen DH, Wu QW, Li XD, Wang SJ, Zhang ZM. SYPL1 overexpression predicts poor prognosis of hepatocellular carcinoma and associates with epithelial-mesenchymal transition. *Oncol Rep*. 2017;38(3):1533-1542.

4. Lanneau D, Brunet M, Frisan E, Solary E, Fontenay M, Garrido C. Heat shock proteins: essential proteins for apoptosis regulation. *J Cell Mol Med*. 2008;12(3):743-61.

5. Foster CS, Dodson AR, Ambroisine L, et al. Hsp-27 expression at diagnosis predicts poor clinical outcome in prostate cancer independent of ETS-gene rearrangement. *Br J Cancer*. 2009;101(7):1137-44.

6. Albany C, Hahn NM. Heat shock and other apoptosis-related proteins as therapeutic targets in prostate cancer. *Asian J Androl*. 2014;16(3):359-63.

7. Chatterjee S, Burns TF. Targeting Heat Shock Proteins in Cancer: A Promising Therapeutic Approach. *Int J Mol Sci*. 2017;18(9)

8. Yu EY, Ellard SL, Hotte SJ, et al. A randomized phase 2 study of a HSP27 targeting antisense, apatorsen with prednisone versus prednisone alone, in patients with metastatic castration resistant prostate cancer. *Invest New Drugs*. 2018;36(2):278-287.

9. Osswald M, Jung E, Sahm F, et al. Brain tumour cells interconnect to a functional and resistant network. *Nature*. 2015;528(7580):93-8.

10. Zhang F, Ying L, Jin J, et al. GAP43, a novel metastasis promoter in non-small cell lung cancer. *J Transl Med*. 2018;16(1):310.

11. Zhao L, Lee BY, Brown DA, et al. Identification of candidate biomarkers of therapeutic response to docetaxel by proteomic profiling. *Cancer Res*. 2009;69(19):7696-703.

12. O'Connell K, Prencipe M, O'Neill A, et al. The use of LC-MS to identify differentially expressed proteins in docetaxel-resistant prostate cancer cell lines. *Proteomics*. 2012;12(13):2115-26.

13. Marin-Aguilera M, Codony-Servat J, Kalko SG, et al. Identification of docetaxel resistance genes in castration-resistant prostate cancer. *Mol Cancer Ther*. 2012;11(2):329-39.

14. Naor D, Nedvetzki S, Golan I, Melnik L, Faitelson Y. CD44 in cancer. *Crit Rev Clin Lab Sci*. 2002;39(6):527-79.

15. Prochazka L, Tesarik R, Turanek J. Regulation of alternative splicing of CD44 in cancer. *Cell Signal*. 2014;26(10):2234-9.

16. Cabrera MC, Hollingsworth RE, Hurt EM. Cancer stem cell plasticity and tumor hierarchy. *World J Stem Cells*. 2015;7(1):27-36.

17. Morath I, Hartmann TN, Orian-Rousseau V. CD44: More than a mere stem cell marker. *Int J Biochem Cell Biol*. 2016;81(Pt A):166-173.

18. Puhr M, Hoefer J, Schafer G, et al. Epithelial-to-mesenchymal transition leads to docetaxel resistance in prostate cancer and is mediated by reduced expression of miR-200c and miR-205. *Am J Pathol*. 2012;181(6):2188-201.

19. Wang L, Zuo X, Xie K, Wei D. The Role of CD44 and Cancer Stem Cells. *Methods Mol Biol*. 2018;1692:31-42.

20. Nagano O, Saya H. Mechanism and biological significance of CD44 cleavage. *Cancer Sci*. 2004;95(12):930-5.

21. Kato T, Mizutani K, Kawakami K, Fujita Y, Ehara H, Ito M. CD44v8-10 mRNA contained in serum exosomes as a diagnostic marker for docetaxel resistance in prostate cancer patients. *Heliyon*. 2020;6(7):e04138.

22. Lai CJ, Lin CY, Liao WY, Hour TC, Wang HD, Chuu CP. CD44 Promotes Migration and Invasion of Docetaxel-Resistant Prostate Cancer Cells Likely via Induction of Hippo-Yap Signaling. *Cells*. 2019;8(4)
